# Preparation and In-Vitro Assessment of Hierarchal Organized Antibacterial Breath Mask Based on Polyacrylonitrile/Silver (PAN/AgNPs) Nanofiber

**DOI:** 10.3390/nano8070461

**Published:** 2018-06-25

**Authors:** Davood Kharaghani, Muhammad Qamar Khan, Amir Shahzad, Yuma Inoue, Takayuki Yamamoto, Selene Rozet, Yasushi Tamada, Ick Soo Kim

**Affiliations:** 1 Nano Fusion Technology Research Group, Division of Frontier Fibers, Institute for Fiber Engineering (IFES-), Interdisciplinary Cluster for Cutting Edge Research (ICCER), Shinshu University, Tokida 3-15-1, Ueda, Nagano 386-8567, Japan; kharaghani66@gmail.com (D.K.); qamarkhan154@gmail.com (M.Q.K.); 15f2020e@shinshu-u.ac.jp (Y.I.); 18fs326j@shinshu-u.ac.jp (T.Y.); 2 Faculty of engineering, National Textile University Faisalabad, Faisalabad 37610, Pakistan; Amir_textilian@hotmail.com; 3 Faculty of Textile Science and Technology Bioresource and Environmental Science, Shinshu University, Tokida 3-15-1, Ueda, Nagano 386-8567, Japan; 15st154e@shinshu-u.ac.jp (S.R.); ytamada@shinshu-u.ac.jp (Y.T.)

**Keywords:** antibacterial mask, in situ synthesis, nanofibers membrane, silver nanoparticles, nanocomposites

## Abstract

In this report, we designed and synthesized polyacrylonitrile/silver (PAN/AgNPs) nanofibers via an in-situ method to obtain a washable with high-dispersed silver nanoparticles membrane to form the hierarchically organized antibacterial mask to prevent the two-way effect of bacteria from person to environment and environment to person. For this objective, the electrospun PAN nanofibers were stabilized via the heating method. Different amounts of AgNPs were loaded into the PAN nanofibers by using silver nitrate and sodium hydroxide solutions. The basic results showed that AgNPs was homogenously loaded in PAN nanofiber matrixes. Furthermore, the release profile based on two-stage release theory showed that when the negligible amount of AgNPs was loaded into the nanofibers, the release significantly decreased, whereas antibacterial activity increased. The greatest potential antibacterial activity of the lowest amount of AgNPs showed controllable AgNPs release from PAN nanofibers that has a direct relationship with the washability and could promote the application of the produced product.

## 1. Introduction

Over the last 50 years, antibiotic resistance has been a major health concern in the world since bacteria progressively adapted to antibiotics [1]. Consequently, scientific efforts have been made to develop new compounds against bacteria and fungi [2], mainly in wound dressings [3] and textiles [4]. Textile is one of the most important necessities of daily life. Therefore, textile materials are a suitable place for growing microorganisms such as fungi, algae, and bacteria, especially in medical textiles such as surgical gowns, gloves, and masks [5]. Consequently, functional activity and structure of materials related to antibacterial properties are very important [6].

However, many antibacterial additives such as phenols, inorganic salts, organometallics [5], organic material like honey [7], and metallic nanoparticles, as well as zinc oxide, titanium oxide [8], and silver nanoparticles (AgNPs) [9] with chemical characteristics [10,11,12] have been used in electrospun nanofibers for wound dressings. However, silver nanoparticles among the antibacterial materials showed the greatest potential to be employed in various fields such as biomedical textiles [13] or electronics [14], thanks to their unique properties in electroconductivity and antibacterial activity [15]. In recent years, some studies have been done to form a breathing mask to control surrogates of viral particles [16] and protective surgical face masks which impact [17] human thermoregulation through impairment of convention, evaporation, and radiation processes [18,19].

However, the researchers did not focus on the stabilized AgNPs in the nanofiber structures to produce an antibacterial mask by hierarchically organizing the nanofiber membrane to control the AgNPs release by chemical bonding. Nanofibers showed the ability to be made into an ultrafine solid with continuous fibers and a controlled surface and molecular structures for antibacterial properties [7]. In addition, a wide range of polymers has been employed as primary materials with specific arrangements for modification of the nanofiber surface with biochemical materials [8]. The electrospun nanofibers have great potential for a controlled drug delivery system. Release profile plays a critical role in promoting the applications of products due to the toxicity of nanomaterials and their effect on the environment via washing effluent. The textile product, as well as socks, could release 1360 μg-Ag/g-sock and leach as much as 650 μg of silver into 500 mL of distilled water over 24 h [20].

Therefore, we attempted to fabricate a polyacrylonitrile/silver (PAN/AgNPs) nanofibers composite membrane for a washable antibacterial mask by the hierarchical arranging of layers. PAN/AgNPs nanofibers composite membrane, due to its two-way protection against bacteria and dust, was chosen as the main component of this research. The morphology of nanofiber and nanoparticles has been studied by scanning electron microscope and transmission electron microscope (TEM). Attenuated total reflection (ATR) and X-ray photoelectron spectroscopy (XPS) have been employed for chemical characterization and confirming the presence of AgNPs in the nanofibers. Inductively coupled plasma mass spectrometry (ICP-MS) was used for direct measurement of the ppm amount of AgNPs upon release and loaded amount in nanofibers. Agar disc diffusion method was chosen for antibacterial activity test.

## 2. Materials and Methods

### 2.1. Materials

Polyacrylonitrile (PAN) with (average Mw 150,000) dimethyl sulfoxide (99.9%) (DMSO) was purchased from Sigma Aldrich Corporation USA, *N*,*N*-Dimethylformamide (DMF) (99.59%). Silver nitrate (AgNO_3_, 99.8%), sodium hydroxide (NaOH, 97%), and hydrochloric acid (HCl, 35–37%) were purchased from Wako Pure Chemical Industries, Ltd. (Nagano Kin, Nagano, Japan) and deionized water was used.

### 2.2. PAN/AgNPs Preparation

**Figure 1:**
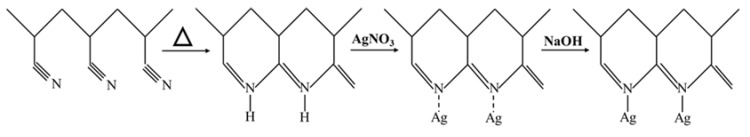
The mechanism of chemical reaction of AgNPs with PAN nanofibers.

The 10% *w*/*w* PAN solution was prepared in DMF with stirring for 5 h at room temperature. The spinning solution was loaded in a plastic syringe with a tip diameter of 0.6 mm and a copper wire was chosen as the positive electrode. The distance from the capillary tip to the collector was kept at 13 cm and then supplied with a voltage of 12 kV. The nanofibers were formed without beads at room temperature and in 45% humidity. The resultant nanofibers mat was stabilized via heating at 250 °C for 2 h in an air atmosphere. The PAN/AgNPs was prepared by dipping the nanofibers mat in a silver nitrate solution, followed by dipping in an alkaline solution. For this purpose, 1 gL silver nitrate solution was prepared and the PAN nanofibers mat was supplied directly to 40 mL of silver nitrate solution for 12 h. Then, PAN nanofibers containing the silver ions were rinsed with deionized water and subjected to 40 mL sodium hydroxide solution of 1 g/L for 12 h. Nanofibers were placed in deionized water for 15 min and shaken to wash out the extra sodium hydroxide and silver ions. This cycle was repeated for three times to prepare the different amounts of silver nanoparticles through the PAN nanofibers. Finally, the obtained PAN/AgNPs was dried at 30 °C and used for characterization. The mechanism of chemical reaction of AgNPs with PAN nanofibers [21] is shown in Figure 1.

### 2.3. SEM & TEM Characterizations

The morphology of the nanofibers was observed by using scanning electron microscopy (SEM, S-3000N, Hitachi Co., Nagano Kin, Nagano, Japan) with accelerating voltage of 12 kV. The dispersion of nanoparticles on nanofibers was studied by transmission electron microscopy (TEM, 2010 Fas TEM, JEOL, Nagano, Japan) with an accelerating voltage of 200 kV that was used to analyze the dispersion of AgNPs on the nanofibers. The average diameter and size distributions of the nanofibers and AgNPs were determined from SEM and TEM micrographs by image analysis software (image J, version 1.49). 

### 2.4. ATR Characterizations

Attenuated total reflectance (ATR) spectroscopy (DuraSamplIR II, Smiths Detection Company, London, UK) of the nanofibers was recorded between the wavelengths of 450 and 4000 cm^−1^ with a resolution of 4 cm^−1^ and the addition of 128 scans at room temperature. The samples in solid and dry form were directly supplied to ATR.

### 2.5. XPS Characterizations

X-ray photoelectron spectroscopy (XPS) was conducted on a Shimadzu-Kratos AXIS-ULTRA HAS SV (Shimadzu Co., Ltd. Nagano, Japan) by using an Al X-ray source set at 10 kV and 15 mA to confirm the presence of AgNPs in the nanofibers. 

### 2.6. Release Profile Study by Inductively Coupled Plasma Mass Spectrometry (ICP-MS)

The amount of loaded and released AgNPs from nanofibers was directly measured by inductively coupled plasma mass spectrometry (ICP-MS) (SHIMADZU/ICPS-1000 IV, Nagano, Japan). The release profile over a period of 72 h was studied by dipping 0.1 g of nanofibers in a plate containing 20 mL of deionized water by shaking at 28 °C. During the release, 2 mL of solution was removed and exchanged by deionized water at precise times (0.25, 0.5, 1, 2, 4, 8, 12, 24, 36, 48, 60, and 72 h). The solution containing AgNPs, which was removed from the source each time, was subjected to ICP-MS spectrometry for measurement of AgNPs released into the deionized water. The amount of silver loaded into nanofibers was measured by dissolving the 0.01 g nanofibers in 5 mL DMSO and diluting the 100 µL of the obtained clear solution with 900 µL deionized water and supplied to ICP-MS spectrometry. The release profile was presented as a percentage based on the below formula:

(1) $$Release\;\left(\%\right)=\frac{Mt}{Mi}\times 100$$

where ***Mt*** is the amount of AgNPs released after 72 h, ***Mi*** is the total amount of AgNPs loaded in PAN nanofibers, and all calculations are done based on using 1 g nanofibers.

### 2.7. Microbiological Experiment

The antibacterial properties of PAN nanofibers containing different amounts of silver nanoparticles were examined with two bacterial strains (*Staphylococcus* and *Pseudomonas*) with the disc diffusion method. In this method, *Pseudomonas* and *Staphylococcus* were used as model bacteria. In this method, the bacteria are cultured in tryptic soy broth liquid medium and incubated overnight at 35 °C. Bacteria from the prepared source were seeded by dispersing TSB containing the bacteria on the surface of nutrient agar plates and nanofibers placed on the cultured diffusion plates for each strain and incubated overnight at 35 °C. To measure the quantified antibacterial properties of samples, colony formation unit method used base on ISO 22196. Briefly, the cell concentration of 10^5^ per mL was prepared by using each strain bacteria in PBS 1X after seeding in tryptic soy broth. The samples were placed in each solution under shaking at 35 °C for 24 h. The series of dilutions were prepared using PBS 1X and 20 µL of each diluted solution containing bacteria placed on agar plates and incubated for almost 24 h at 35 °C. The percentage of viable bacteria was calculated using the formula below:

(2) $$\mathrm{CFU}\text{\%}=\;\frac{{\mathrm{CFU}}_{S}}{{\mathrm{CFU}}_{C}}\;\times 100$$

where CFU_S_ shows the colony formation unit for samples and CFU_C_ is assigned to colony formation unit for control. 

### 2.8. Toxicity 

The toxicity of nanofibers in the presence of AgNPs was evaluated by using direct contact based on ISO 10993-5. Briefly, cells were seeded evenly over the surface of each plate and incubated at 37 °C until the cells covered the whole surface. The samples were then placed on the cells layer in the center of the plates and the culture medium was replaced. In order to determine the toxicity in accordance with grade 0 (nontoxic) to grade 4 (severe toxic) evaluation, trypan blue was added after 24 h in each plate and observed by microscope.

### 2.9. Tensile Strength 

The mechanical properties of the final composite nanofiber with one cycle loaded AgNPS before and after wash during 72 h were assessed using a universal testing machine (TENSILON RTC1250A, A&D Company, Ltd., Nagano, Japan) under a cross-head speed of 1 mm/min at room temperature. In accordance with ASTM D-638, the nonwoven nanofibers were prepared in the shape of a dumbbell to test their tensile strength. Stress and strain were calculated by Formulas (3) and (4) as mentioned below:

(3) $$\;Stress\;\left(Strength\right)=\frac{Force\left(N\right)}{Area\;\left(width*thichness\right)}$$

(4) $$\;Strain\;\left(Elongation\right)=\frac{Change\;in\;length\left(\Delta l\right)}{Initial\;length\left(l\right)}\;\times 100$$

## 3. Results and Discussion

### 3.1. Morphology of Nanofibers

**Figure 2:**
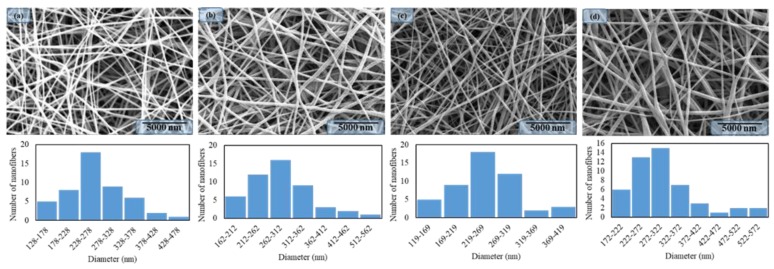
SEM images & nanofibers diameter distributions of Neat PAN nanofibers: (**a**), PAN/AgNPs nanofibers having one dipping cycle: (**b**), PAN/AgNPs nanofibers having two dipping cycles: (**c**), and PAN/AgNPs nanofibers having three dipping cycles: (**d**).

In order to investigate the morphology of PAN/AgNPs nanofibers, SEM was employed to observe the composite nanofibers as shown in Figure 2. It was observed that all PAN/AgNPs nanofibers were prepared without beads and appreciable surface morphology. On the other hand, size distributions and the average nanofiber diameters were calculated by Image J software and it was analyzed that the average diameter was affected by the load of the AgNPs to the PAN nanofibers. The average diameter of the PAN nanofibers without AgNPs was 263 nm and it was changed to 290, 250, and 302 nm, respectively, as the number of dipping cycles was increased. It was observed that cycle two had a size distribution near to pure PAN nanofibers that may have happened due to choosing the nanofibers randomly. From the morphology study, it was concluded that the number of dipping cycles had a significant effect on the size distributions of nanofibers.

**Figure 3:**
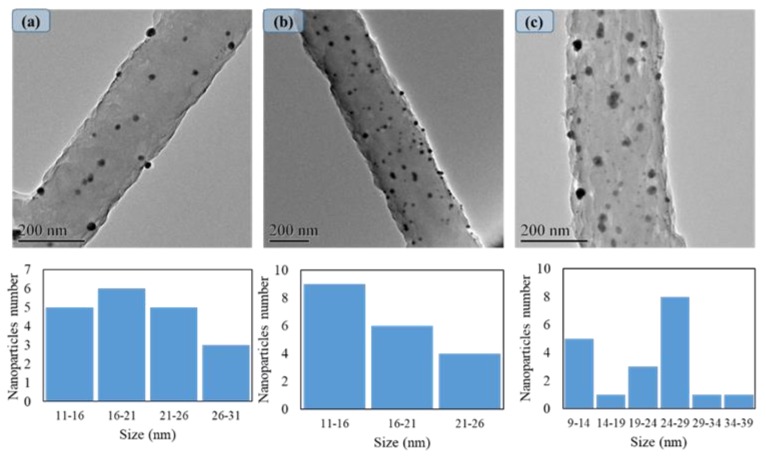
TEM images & size distributions of PAN/AgNPs nanofibers one cycle loaded: (**a**), PAN/AgNPs nanofibers two cycles loaded: (**b**), and PAN/AgNPs nanofibers three cycles loaded: (**c**).

In order to investigate the AgNPs morphology, TEM was used to analyze the dispersion of nanoparticles into the nanofibers as shown in Figure 3. It was observed that there was a uniform dispersion of nanoparticles on the nanofibers with different size distributions. These results confirmed that the number of dipping cycles did not have a significant effect on the dispersion of nanoparticles, and dispersion of AgNPs was also uniformly increased by increasing the dipping cycles. On the other hand, the size distributions of AgNPs showed that the average size for AgNPs did not have significant differences among the cycles and it was calculated at 20 nm, 18 nm, and 21 nm, respectively, by increasing the cycles. However, as shown in Figure 3c, the maximum size for AgNPs in the third cycle was around 39 nm. It seems that increasing the number of dipping cycles of PAN/AgNPs could cause agglomeration and have a significant effect on the appearance of the bigger particle.

### 3.2. Study of ATR-FTIR Spectra

**Figure 4:**
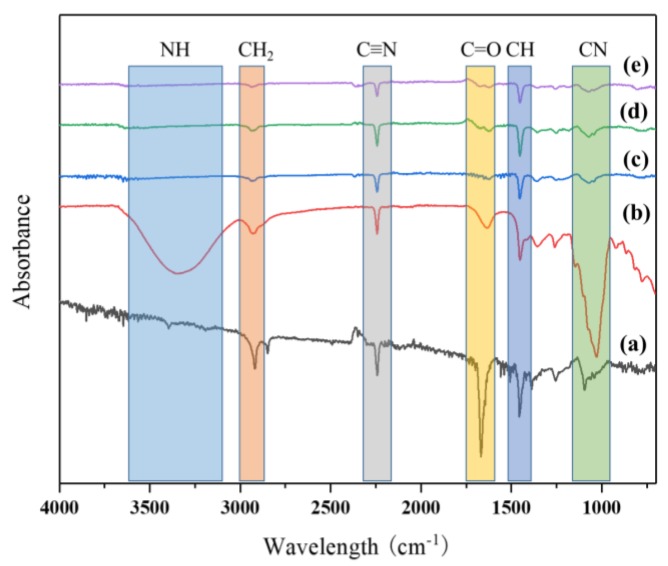
ATR Spectra of PAN nanofibers: (**a**), stabilized neat PAN nanofibers: (**b**), PAN/AgNPs nanofibers one cycle loaded: (**c**), PAN/AgNPs nanofibers two cycles loaded: (**d**), PAN/AgNPs nanofibers three cycles loaded: (**e**).

In order to investigate the chemical structure of the functional groups of a pure PAN nanofiber mat, a stabilized pure PAN nanofiber mat and a PAN nanofiber mat containing the different amounts of AgNPs were studied by using ATR-FTIR spectroscopy as shown in Figure 4. In the ATR-FTIR graph for neat PAN nanofibers before stabilization, CH stretching peak appeared at 2914 cm^−1^ and C≡N band was around 2241 cm^−1^ stretching, and a strong signal at 1666 cm^−1^ appeared due to the C=O band. After stabilization, obvious peaks were observed about C≡N and C=O changed and an extensive band at 3344 cm^−1^ was related to symmetric stretching of the NH group formed, and in comparison to Zhang et al., it seems that this peak appeared due to stabilization of PAN nanofibers [22]. It was observed that this peak due to the presence of aliphatic CH and CH_2_ vibration appeared in 1634, 1452, and 1351 cm^−1^. However, the presence of a strong peak at 1031 cm^−1^ was attributed to the C-N functions group of PAN nanofibers [23]. 

On the other hand, with the in situ synthesis of the AgNPs on the nanofibers, the intensity of strong peaks at 3344 cm^−1^ and 1031 cm^−1^ decreased significantly. It seems that this phenomenon appeared due to the reaction between the Ag ions with NH and the C-N group through the nanofibers [21]. However, the presence of NH and C-N groups created the active sites for absorbing Ag ions with a negative charge and AgO synthesized by reduction of Ag ions in the presence of the PAN nanofibers matrix. However, the functional groups related to PAN nanofibers at 2928 cm^−1^, 2243 cm^−1^, and 1452 cm^−1^, respectively, due to the presence of CH_2_ stretching, C≡N, and CH_2_ vibrations, appeared without any changes in the various amounts of AgNPs and it seems that the presence of AgNPs did not have any effect on changing the chemical structure of PAN nanofibers. The chemical reaction between the AgNPs with PAN nanofibers occurred flowing by covalence bonding with NH and C-N groups as shown in Table 1

### 3.3. Study of XPS Spectra

**Figure 5:**
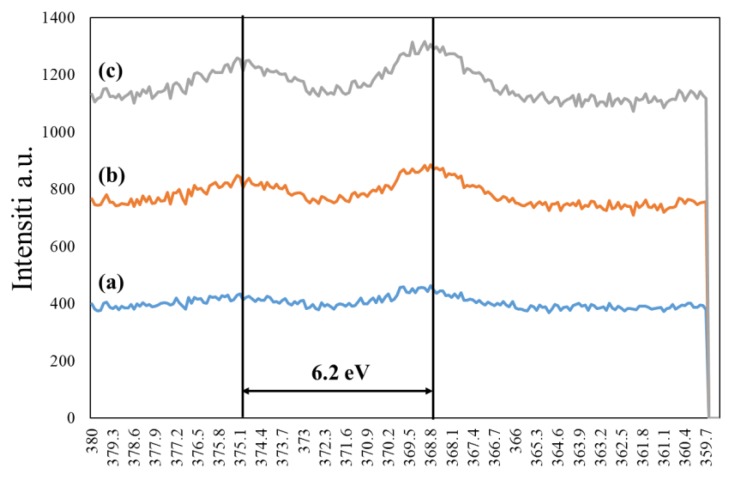
XPS study of PAN/AgNPs nanofibers one cycle loaded: (a), PAN/AgNPs nanofibers two cycles loaded: (b), a PAN/AgNPs nanofibers three cycles loaded: (c).

In order to confirm the presence of silver nanoparticles on the nanofibers, the XPS spectra were studied and the resultant graph is shown in Figure 5. The Ag3d peaks for all samples appeared at 368.6 eV and 374.8 eV, respectively, due to assigning as Ag3d_5/2_, Ag3d_3/2_, and the slitting between core levels calculated by 6.2 eV that indicate a normal state of AgNPs. However, the peaks related to AgNPs have been compared with literature that focused on synthesizing the AgNPs with the immersed polymer matrix [24]. The result showed that the AgNPs was synthesized successfully and the intensity increased by increasing the dipping cycles on the PAN nanofibers, and the presence of the PAN nanofiber matrix did not have a significant effect on the chemical structure of AgNPs. 

### 3.4. The Release Profile of Silver into the Water

**Figure 6:**
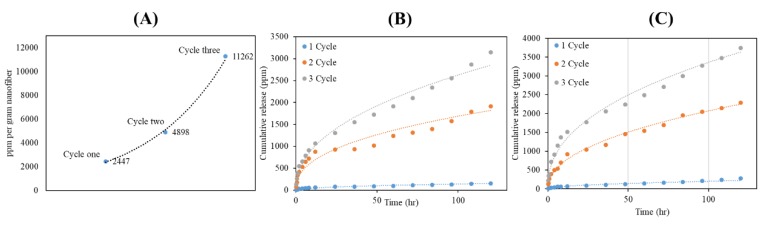
The amount AgNPs loaded (ppm) in one gram PAN nanofibers: (**A**), the release profile of PAN/AgNPs nanofibers at 28 °C: (**B**), and release profile of PAN/AgNPs nanofibers at 37 °C: (**C**).

The amount of loaded AgNPs in 1 g PAN nanofibers was calculated as 2447, 4898, and 11,262 ppm for the cycles 1, 2, and 3, respectively, as shown in Figure 6A and Table 2. Figure 6B,C shows the release behavior of AgNPs from PAN nanofibers for five days within two different temperatures. Recently, much research has been attempted to control the release of AgNPs from nanofibers, but the research does not demand the properties for preparing the washable nanofibers containing silver nanoparticles [25,26]. However, the theoretical comparison of results for two-stage with a limited desorption release which matched with one cycle loaded AgNPs into the PAN nanofibers in comparison to Zupančič et al. has been studied. Two-stage release based on Zupančič et al.’s research defines a process where the embedded drug is released from nanopores of the individual nanofibers or from the outer surface of these fibers in contact with water [27]. From the results, it was confirmed that the release profile would be improved by the composition of nanoparticles with nanofibers. The release of AgNPs may happen from the mat nanofibers and be collected between nanofiber pores. This release continued from the pores to the environment, which was translated by an improvement of the release profile. However, the release profiles of cycle 2 and cycle 3 revealed sigmoid shapes uncharacteristic of the two-stage in comparison to the research of Alexander L. Yarin et al., which studied and described the release profiles of the two-stage desorption theory [28]. Furthermore, the results showed the ability of one cycle loaded with AgNPs to control the leached nanoparticles to deionized water during 120 h thanks to its ability to control the amount of release, which had a direct relation with washability, and it was around 6.19% and 11.24% for 28 °C and 37 °C, respectively, of loaded AgNPs. However, the release profile for both temperatures at 28 °C and 37 °C in comparison to previous literatures showed that the in situ method for AgNPs loading in PAN nanofibers had a significant effect on controlled release and matching the release profile with two-stage theory.

### 3.5. Antibacterial Activity

The antibacterial efficiency of the PAN/AgNPs nanofibers was examined by agar diffusion method. In this method, *Pseudomonas* and *Staphylococcus* bacteria were used as model bacteria. Mishra S. K. et al. in 2016 showed that the antibacterial properties of silver nanoclusters which synthesized the AgNCs by in situ method had toxicity around 56% at the maximum amount of AgNCs [29]. However, G. Aleksander et al. evaluated the biocompatibility and antibacterial properties of poly (l-lactide-*co*-glycolide)/AgNPs. Herein, they concluded that 3 wt % AgNPs incorporated with poly (l-lactide-*co*-glycolide) has good biocompatibility despite this concentration showing weak antibacterial results [30].

**Figure 7:**
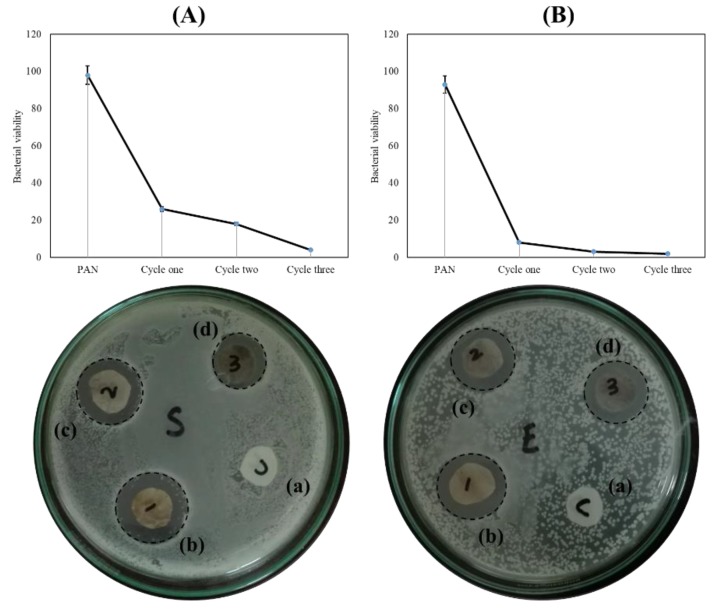
The antibacterial result of PAN/AgNPs nanofibers Staphylococcus: (**A**) and Escherichia-coli: (**B**). (Pure PAN nanofiber: (a), one cycle: (b), two cycles: (c), and three cycles: (d)).

The results of antibacterial efficiency are shown in Figure 7. The results show that the treated nanofibers with AgNPs have an effective antibacterial activity against *Pseudomonas* and *Staphylococcus* bacteria but it also shows that AgNPs presence in the nanofiber matrix has more effect against *Pseudomonas* than against *Staphylococcus*. When the numbers of immersing cycles were increased, the antibacterial properties were also increased as shown in Figure 7. The PAN/AgNPs nanofibers with three cycles of loading AgNPs had great potential for use, with an additional layer for preparing the antibacterial breath mask. The results from antibacterial activity showed that direct interaction between PAN nanofibers containing AgNPs and bacteria may lead to the zones of inhibition surrounding the nanofibers, going proportionally greater with the increase of amount of AgNPs. However, the increase of zones of inhibition showed the greatest potential of antibacterial properties for PAN nanofibers when the amount of AgNPs was the highest [31,32,33]. Results from quantified antibacterial properties of samples showed that the amount of release had a direct effect on the viability of bacteria. However, the disc diffusion method showed different behavior from the colony formation method, possibly resulting from a pool of AgNPs in a limited area in the disc diffusion method while the released AgNPs diluted and dispersed in the solution. Furthermore, Swarnali Maiti et al. showed in their research of antimicrobial activities of silver nanoparticles that when a 50 µg/mL amount of AgNPs is increased to 80 µg/mL, the antibacterial properties significantly decreased. The results from quantitative and qualitative antimicrobial measurement showed that increasing the concentration of AgNPs did not help to improve antibacterial properties [34].

**Figure 8:**
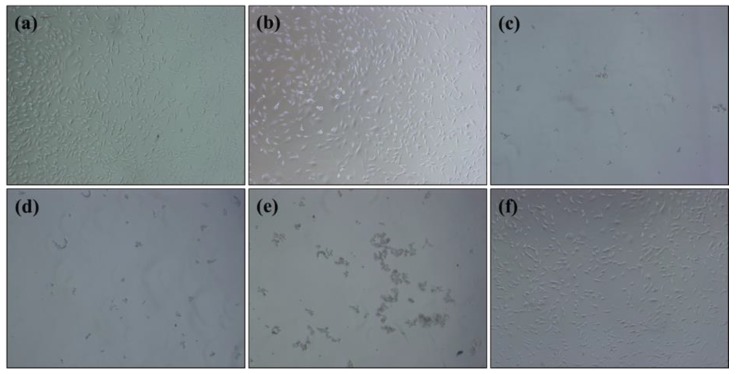
The toxicity evaluation of pure PAN nanofiber: (**a**), with one cycle AgNPs loaded: (**b**), with two cycles AgNPs loaded: (**c**), with three cycles AgNPs loaded: (**d**), positive control: (**e**), and negative control: (**f**).

The results as displayed in Figure 8 showed that the presence of large amounts of AgNPs caused the toxicity of nanofibers while the pure PAN nanofibers did not show any toxicity in contact with cells. However, the sample with one cycle loaded AgNPs had the minimum rate of toxicity less than grade 1 with appropriate antibacterial properties. Samples with two and three cycles loaded AgNPs had the maximum toxicity (grade 4). From the results obtained, the sample with one cycle loaded AgNPs had suitable biocompatibility for use as an antibacterial layer for preparing the breath mask. 

### 3.6. Mechanical Properties 

**Figure 9:**
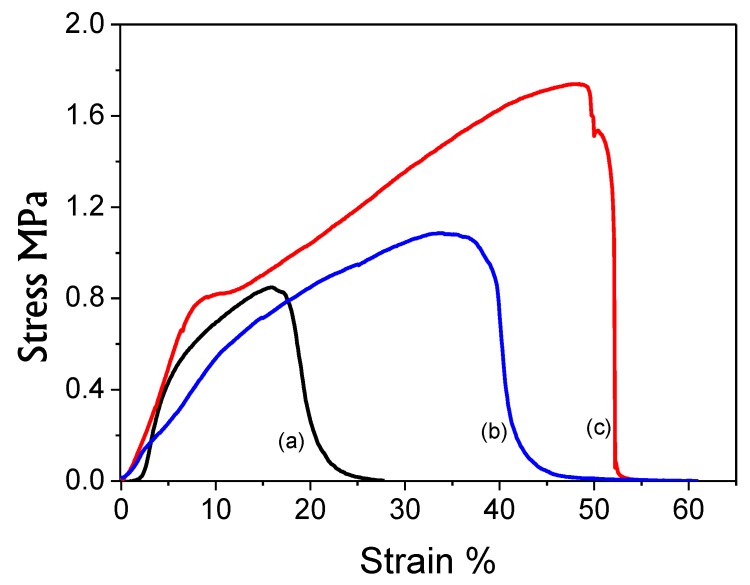
The tensile strength of PAN nanofibers: (**a**), one cycle loaded AgNPs to PAN nanofibers after 72 h washing: (**b**), and one cycle loaded AgNPs to PAN nanofibers: (**c**).

In order to investigate the mechanical strength behavior of the PAN nanofibers, PAN/AgNPs nanofibers and washed PAN/AgNPs nanofibers with one cycle loaded AgNPs were studied as shown in Figure 9. It was analyzed that neat PAN nanofibers had the maximum tensile strength 0.9 MPa with 18% elongation while PAN/AgNPs nanofibers had 1.8 MPa tensile strength and 52% elongation, which was larger than neat PAN nanofibers. However, results showed that AgNPs presence had a significant effect on tensile strength of PAN nanofibers. Therefore, mechanical strength behavior of PAN/AgNPs nanofibers after 72 h washing in deionized water showed that PAN/AgNPs nanofibers had maintained the appreciable mechanical properties with tensile strength around 1.1 MPa with 40% elongation, which was again greater than neat PAN nanofibers as shown in Figure 9. Hence, due to the hierarchical structure of the designed breath mask, PAN/AgNPs nanofibers will support layers of fabrics that cover the PAN/AgNPs nanofibers, while mechanical strength behavior in Figure 9 were reported without any supports.

## 5. Conclusions

Herein, we successfully designed a washable, hierarchically organized, antibacterial mask by in situ synthesis of metallic nanoparticles on PAN/AgNPs nanofibers. On the basis of characterizations, it was concluded that PAN/AgNPs nanofibers, which were fabricated with one cycle loading AgNPs (2247 ppm/gr nanofiber) made ideal membranes for antibacterial properties despite their lowest antibacterial properties in comparison to cycles two and three. It seems that the ability of one cycle loading AgNPs is enough for the preparation of a washable antibacterial mask. The release profile of AgNPs in the 1 gr of PAN nanofibers has been shown to control the release ratio during 120 h that emphasized the ability of this method for preparing washable antibacterial products. Therefore, PAN/AgNPs nanofibers fabricated with one cycle of loading AgNPs had the optimal potential for antibacterial properties and biocompatibility. Results showed this type of membrane is the most suitable use of nanofibers to form the hierarchically organized antibacterial breath mask with appropriate biocompatibility (less than grade 1) to prevent the two-way effect of bacteria from person to environment and environment to person. The tensile strength results showed loading silver nanoparticles increased the mechanical properties of PAN nanofibers even after 120 h washing with deionized water. Hence, this proposed antibacterial breath mask would fulfill the stated and implied needs of customers.

## Figures and Tables

**Table 1:** Spectroscopy data

Functional Group	Absorption(s) (cm^−1^)	Notes
CH_2_	2922	CH stretching in CH and CH_2_ groups (a)
C≡N	2243	Stretching (a)
C=O	1666	Stretching (a)
CH	1456	CH blending (a)
CN	1091	CN blending (a)
NH	3340	stretching vibration (b)
CH_2_	2932	CH stretching in CH and CH_2_ groups (b)
C≡N	2243	Stretching (b)
C=O	1641	Stretching (b)
CH	1455	CH blending (b)
CN	1031	CN blending (b)
CH_2_	2922	CH stretching in CH and CH_2_ groups (c), (d) and (e)
C≡N	2247	Stretching (c), (d) and (e)
CH	1456	CH blending (c), (d) and (e)

**Table 2:** The release profile of PAN nanofibers containing AgNPs with different cycles.

Sample	Loaded AgNPs in 1 g Nanofiber	Release Percentage after 120 h 28 °C	Release Percentage after 120 h 37 °C
One cycle loaded	2447 ppm	6.19%	11.24%
Two-cycles loaded	4898 ppm	39.05%	46.73%
Three cycles loaded	11,262 ppm	27.93%	33.23%
